# Early Results of Stereotactic Radiosurgery in Uveal Melanoma and Risk Factors for Radiation Retinopathy

**DOI:** 10.4274/tjo.galenos.2019.78370

**Published:** 2020-06-27

**Authors:** Gökçen Özcan, Ahmet Kaan Gündüz, İbadulla Mirzayev, Kaan Oysul, Hasan Uysal

**Affiliations:** 1 Ankara University Faculty of Medicine, Department of Ophthalmology, Ankara, Turkey; 2 Medicana International Hospital, Clinic of Radiation Oncology, Ankara, Turkey

**Keywords:** Uveal melanoma, stereotactic radiosurgery, radiation retinopathy

## Abstract

**Objectives:**

To report treatment results and complications of stereotactic radiosurgery in uveal malignant melanoma and to identify risk factors for development of radiation retinopathy.

**Materials and Methods:**

This was a retrospective study of 36 patients diagnosed with uveal melanoma between 2014 and 2019. Best corrected visual acuity, funduscopic findings, basal tumor diameter and tumor thickness were recorded at baseline and at follow-up visits at 3-month intervals. The response of tumors to stereotactic radiosurgery and complications were determined.

**Results:**

The mean basal diameter of tumor was 10.2 (range: 4.0-19.4, standard deviation [SD]: ±3.3) mm x 9.7 (range: 4.5-18.0, SD: ±3.3), tumor thickness was 5.1 (range: 2.0-11.0, ±2.4) mm at baseline. The mean follow-up period was 17.2 (range: 6-48, SD: ±10.43) months. The mean visual acuity was 0.5 (SD: ±0.3) logMAR before treatment and 0.6 (SD: ±0.3) logMAR after the mean follow-up period. The most common complications after stereotactic radiosurgery were cataract (38.9%) and radiation retinopathy (27.7%). There was a statistically significant relation between radiation retinopathy development and tumor distance from the optic disc (p=0.04). The rate of eye salvage was 83.3% in this study.

**Conclusion:**

Our short-term results show stereotactic radiosurgery was an effective and sustained treatment modality among the other eye conservation therapies.

## Introduction

Uveal melanoma is the most common primary intraocular tumor in adults. Uveal melanoma usually originates from the choroid (85.0%), followed by the ciliary body (10.0%) and iris (5.0%).^1^ In recent years, globe-preserving surgeries have taken the place of enucleation in the treatment of uveal melanoma. In an arm of the Collaborative Ocular Melanoma Study (COMS), the outcomes of patients in the medium tumor group who underwent iodine-125 plaque brachytherapy and enucleation were compared and no significant difference in long-term survival was detected between plaque brachytherapy and enucleation. Melanoma-related mortality rates in the plaque brachytherapy group were reported as 10%, 18%, and 21% at 5, 10, and 12 years, respectively, while these rates were 11%, 17%, and 17%, respectively, in the enucleation group.^3^ Depending on the location and size of the tumor, globe-preserving treatment options include laser photocoagulation, transpupillary thermotherapy, radiotherapy, and tumor excision (endoresection or exoresection). Radiotherapy for uveal melanoma can be delivered as brachytherapy (plaque radiotherapy) or teletherapy (proton beam radiotherapy, stereotactic radiotherapy). Ophthalmic radioactive plaque brachytherapy involves the use of γ-ray-emitting cobalt-60, palladium-103, and iodine-125, in addition to β-particle-emitting ruthenium-106.^4^

In stereotactic radiosurgery (SRS), tumor location is determined by computed tomography (CT) in order to provide the maximum radiation dose to tumor tissue and minimize radiation to healthy tissue. Devices used for SRS include Gamma Knife, linear accelerators (LINAC), and CyberKnife. Gamma Knife has been used as a successful treatment modality for the treatment of uveal melanoma for the past 15 years.^5^ CyberKnife is a LINAC-based, image-guided SRS system that uses noninvasive fixation. The method is non-invasive, effective, and has a safer adverse-effect profile compared to Gamma Knife.^6,7^ The main reasons we preferred CyberKnife for the treatment of uveal melanoma in our series are that stereotactic surgery causes minimal adverse effects to adjacent tissues and that CyberKnife procedures are covered by government health insurance in Turkey according to the communiqué on healthcare practices. Plaque radiotherapy is not covered by government health insurance.

Radiation retinopathy is a chronic and progressive vasculopathy that causes visual morbidity in patients who receive radiation therapy for malignancies of the globe, orbit, and head and neck region. It was first described by Stallard^8^ in 1933. The primary vascular pathology manifests with endothelial cell loss and capillary bed occlusion.^9^ The retinopathy that occurs subsequent to this vascular damage can be observed in the macula and the peripapillary region and/or peripheral retina.^10^ The most common clinical findings include hard exudates, retinal hemorrhages, microaneurysms, telangiectasia, soft exudates, and retinal or optic disc neovascularization. The onset of radiation retinopathy occurs between 6 months and 3 years after radiation therapy.^11^ Attempts have been made in the past to treat radiation retinopathy with laser photocoagulation, hyperbaric oxygen therapy, pentoxifylline therapy, and photodynamic therapy.^12,13,14^ Vascular endothelial growth factor (VEGF) and other inflammatory and vasculogenic factors play a role in the pathogenesis of macular edema and neovascularization.^15^ For this reason, the use of anti-VEGF agents has come to the fore in the treatment of radiation-related macular edema, neovascularization, and papillopathy.

The aim of this study was to determine the early treatment outcomes and adverse effects of SRS and identify risk factors for radiation retinopathy in patients with uveal melanoma.

## Materials and Methods

Ethics committee approval required for the study was obtained from the Clinical Research Ethics Committee of the Ankara University Faculty of Medicine, and the study adhered to criteria of the Declaration of Helsinki. Thirty-six patients who were diagnosed with uveal melanoma and underwent single-fraction SRS with a single dose of 21 gray (Gy) were retrospectively analyzed. Best corrected visual acuity, intraocular pressure (IOP), affected side, tumor location and distance from the optic disc and fovea, tumor base diameter and thickness, tumor pigmentation, and presence of orange pigment and subretinal fluid were evaluated. Tumor diameter and thickness were measured using B-mode ultrasonography and the presence of subretinal fluid was recorded. Fluorescein angiography was performed on tumors in the posterior pole region. As per COMS, tumors less than 2.5 mm thick and 5-16 mm in diameter were classified as small, those 2.5-10 mm thick and less than 16 mm in diameter as medium, and those more than 10 mm thick and over 16 mm in diameter as large. Tumor classification was also done according to the American Joint Committee on Cancer (AJCC, 8^th^ Edition) (Table 1).

**Figure 1:**
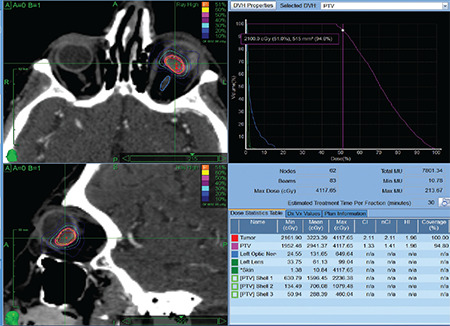
Planning Stereotactic Radiosurgery: Fusion of cranial MRI and cranial CT images of a patient with uveal melanoma in the left eye was performed using the Multiplan (Multiplan® Accuray Incorporated, Sunnyvale, California, USA) contouring software. Using the fused images, PTV was created by adding a 1-mm margin to the tumor. Non-isocentric, non-coplanar planning was done. Dose limits set for the optic nerve and lens were 7 Gy and 3 Gy, respectively. After planning, a 21-Gy treatment was performed with coverage of 100.0% of the tumor and 95.0% of the PTV. The CI was 1.31. The maximum dose to the optic nerve was 6.49 Gy and to the lens was 0.99 Gy MRI: Magnetic resonance imaging, CT: Computed tomography, CI: Conformity index, PTV: Planning target volume, Gy: Gray

The CyberKnife radiosurgery procedure started by making thermoplastic masks for patient immobilization. This was followed by T1- and T2-weighted magnetic resonance imaging (MRI) of the orbit. Immediately after standard retrobulbar anesthesia induction, contrast-enhanced CT images with a slice thickness of 1 mm were obtained. MRI and CT images were superimposed and the gross tumor volume (GTV) was delineated. Clinical target volume (CTV) was obtained by adding a 1-mm margin to the GTV. The planning target volume (PTV) was considered equal to CTV. The lens and optic nerve were marked as critical structures. The 70.0% isodose curve was planned as a single 21 Gy fraction covering 95.0% of the PTV. Dose limits for the lens and optic nerve were set to 2 Gy and 7 Gy, respectively, in cases where the tumor was sufficiently far from the lens and optic nerve. For lesions directly adjacent to the lens or optic nerve, a dose limit was not set for the lens, but it was ensured that the optic nerve received a dose below 12 Gy. The procedure was performed using the CyberKnife device (CyberKnife® MultiPlan® Treatment Planning System, Accuray Incorporated, Sunnyvale, California, USA) (Figure 1).

The patients were examined 1 week after SRS and at 3-month intervals afterwards. Visual acuity, IOP, and fundoscopic examination findings were recorded. Tumor diameter and thickness were measured by B-mode ultrasonography at each visit. Optical coherence tomography (OCT), OCT angiography (OCTA), and fundus fluorescein angiography (FFA) were performed to detect and monitor radiation retinopathy.

The obtained data were analyzed using SPSS (Statistical Package for the Social Sciences) for Windows 15 package software. Descriptive statistics were expressed as mean ± standard deviation (SD) for normally distributed variables and as median (minimum-maximum) for non-normally distributed variables; nominal variables were presented as frequency and percentage. For comparisons between two groups, a t-test was used to evaluate the significance of the differences in means and the Mann-Whitney U test was to evaluate differences in median values. For comparisons between more than two groups, differences in means were evaluated with analysis of variance and differences in median values were evaluated with the Kruskal-Wallis test. The relationship between continuous variables was investigated using Spearman’s correlation coefficient if nonnormally distributed and with Pearson’s correlation coefficient if normally distributed. Results with p<0.05 were regarded as statistically significant.

## Results

**Figure 2:**
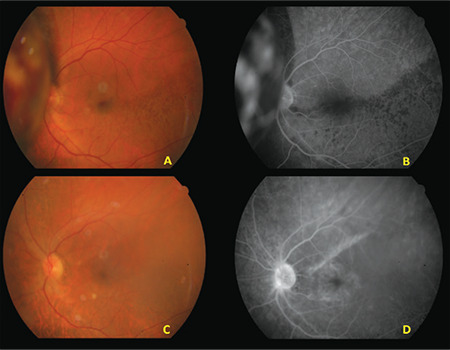
Fundus photograph of a patient with uveal melanoma nasal to the optic disc (A). On fundus fluorescein angiography, leakage over the mass is seen in the late venous phase (B). At 13 months after SRS, fundus photograph shows tumor regression and soft exudates inferior to the fovea (C). In the late venous phase of fluorescein angiography, leakage due to cystoid macular edema and hyperfluorescence of the optic disc due to radiation papillopathy are observed (D) SRS: Stereotactic radiosurgery

Twenty-three (63.9%) of the patients were men and 13 (36.1%) were women. The mean age at diagnosis was 60.5 (range: 28-86, SD): ±14.8) years. In terms of tumor location, 28 tumors (77.8%) were choroidal, 7 (19.4) were ciliochoroidal, and 1 (2.8%) was iridociliochoroidal. Nine (25.0%) of the tumors were amelanotic and 25 (69.4%) had subretinal fluid. The most common tumor location was the temporal macular region. Mean distance from the optic disc was 3.9 (range: 0.0-14.0 mm, SD: ±3.4) mm and mean distance from the fovea was 3.2 (range: 0.0-9.5, SD: ±3.2) mm. The best corrected visual acuity at baseline was 0.5 (SD: ±0.3) logMAR (Table 2). On ultrasonography, uvea melanomas appeared as a dome- or mushroom-shaped, low- to mid-reflective mass. On FFA, the lesion appeared hyperfluorescent starting in the late venous phase. This hyperfluorescence increased in the late phases and appeared as leakage from the lesion surface (Figure 2).

Pre-treatment mean tumor base diameter and mean tumor thickness measured by ultrasonography were 10.2 (range: 4.0-19.4, SD: ±3.3) x 9.7 (range: 4.5-18.0, SD: ±3.3) mm and 5.1 (range: 2.0-11.0, SD: ±2.4) mm, respectively. According to the COMS classification, 31 (86.2%) of the patients had medium tumors, 3 (8.3%) had large tumors, and 2 (5.5%) had small tumors. According to the AJCC TNM classification, 4 cases (11.1%) were T1aN0M0, 8 cases (22.3%) were T2aN0M0, 2 cases (5.5%) were T2bN0M0, 15 cases (41.7%) were T3aN0M0, 4 cases (11.1%) were T3bN0M0, 1 case (2.8%) was T4aN0M0, and 2 cases (5.5%) were T4bN0M0. The mean radiation dose (MRD) applied to the tumors was 2456 cGy (SD: ± 212.6), the MRD to the disc was 164.1 cGY (SD: ± 131.2), and the MRD to the lens was 132.4 cGY (SD: ± 83.5).

The mean follow-up period was 17.2 (range: 6.0-48.0, SD: ±10.4) months. At the end of the mean follow-up, mean tumor base diameter and thickness were 10.8 (range: 4.5-20.0, SD: ±3.6) x 9.8 (range: 4.5-18.0, SD: ±3.1) mm and 5.1 (range: 2.0-11.0, SD: ±2.4) mm, respectively (p=0.001). Best corrected visual acuity at the end of mean follow-up was 0.6 (SD: ±0.3) logMAR (p=0.2). Complications that occurred after SRS included cataract, radiation retinopathy, radiation maculopathy, radiation papillopathy, glaucoma, and scleral thinning. Fourteen patients (38.9%) developed radiation-induced cataract during the follow-up period. The most common cataract type was posterior subcapsular cataract. There was a significant relationship between cataract formation and the dose to the lens during radiosurgery (p=0.04). In 60.0% of patients with cataracts, the tumor was located adjacent to the optic disc.

**Figure 3:**
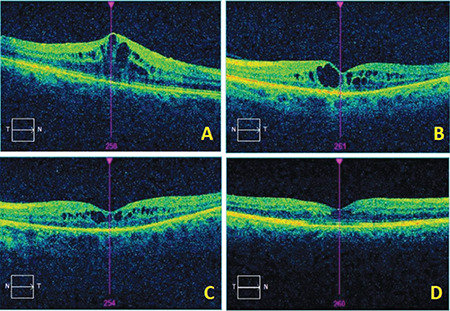
At 13 months after SRS, OCT reveals cystoid macular edema in a patient whose fluorescein angiography shows macular leakage (A). Regression of the macular edema was observed after 2 monthly injections of aflibercept (B). After the third dose of aflibercept, the intraretinal cysts diminished in size (C) and after the fifth dose of aflibercept, there was substantial improvement of the cystoid edema in the macula (D) SRS: Stereotactic radiosurgery, OCT: Optical coherence tomography

Ten (27.7%) of the patients developed radiation retinopathy retinopathy based on fundoscopic findings. Macular changes were confirmed with OCT. The mean time to develop radiation retinopathy was 12 (SD: ±4) months. The relationship between tumor distance from the disc and the development of radiation retinopathy was found to be statistically significant (p=0.001). Development of radiation retinopathy was not significantly associated with MRD to the tumor (p=0.53), tumor thickness (p=0.69), or tumor distance from the fovea (p=0.55). Of the 10 eyes that developed radiation maculopathy, 8 were given anti-VEGF therapy. Five eyes received ranibizumab injections, 2 eyes received aflibercept injections, and 1 eye received bevacizumab injections. The mean number of injections was 8.5 (SD: ±5.7) (Figure 3). The mean visual acuity of the patients treated with intravitreal injections was 0.8 (SD: ±0.1) logMAR pre-treatment and 0.6 (SD: ±0.1) with LogMAR post-treatment (p=0.07). Three patients (8.3%) had radiation papillopathy, 2 (5.6%) had secondary glaucoma, and 1 (2.8%) had scleral thinning. Of the 5 eyes (14.0%) that showed regrowth on ultrasonography and 2 eyes (5.6%) that developed neovascular glaucoma (7 eyes in total), 5 underwent enucleation and 2 underwent endoresection. Of the patients who underwent endoresection, one had subsequent enucleation. The globe preservation rate was 83.3%.

**Figure 4:**
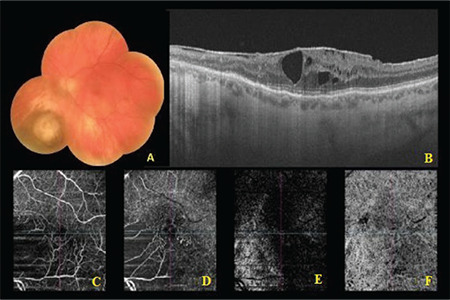
Color fundus photograph of a patient who underwent 21 Gy SRS and developed radiation maculopathy after 11 months. The patient received 17 doses of ranibizumab (A). Swept-source OCT image showing ERM and cystoid macular edema. Atrophy of the outer retina and RPE in the nasal fovea with associated reverse shadowing (B). OCTA images show areas of capillary dropout in the superficial (C) and deep (D) capillary plexuses, non-flow areas due to cystoid macular edema, and ERM-induced vascular traction. The choriocapillaris vasculature is visible due to RPE atrophy in the outer retina (unmasking) (E). Artifacts consisting of cystoid spaces and signal void areas due to shadowing are observed in the choriocapillaris layer (F) SRS: Stereotactic radiosurgery, Gy: Gray, RPE: Retinal pigment epithelium, OCT: Optical coherence tomography, OCTA: Optical coherence tomography angiography, ERM: Epiretinal membrane

In the patients who developed radiation maculopathy, OCT revealed intraretinal edema, epiretinal membrane (ERM), and subretinal fluid (Figure 3a-4b). OCTA of these patients demonstrated an enlarged and irregular foveal avascular zone, nonperfusion, and microaneurysms in the superficial and deep capillary plexuses (Figure 4). FFA revealed areas of nonperfusion around the tumor and cystoid macular edema (Figure 2d).

## Discussion

CyberKnife is a LINAC-based, robot-controlled radiosurgery system. With the possibility of radiation rays coming from an almost infinite number of angles, it only targets tumor tissue and aims to preserve healthy radiosensitive tissue. Since its introduction, CyberKnife has become an alternative to Gamma Knife. The main drawbacks of the Gamma Knife system were the invasive immobilization via the rectus muscles, the need for long-lasting general anesthesia/sedation, and the unfavorable adverse-effect profile due to the optimal dose for one-time therapy being up to 40 Gy. Haas et al.^16^ reported radiation retinopathy in 84.0% and neovascular glaucoma in 47.0% of patients after single fraction Gamma Knife treatment (50 median Gy) for choroidal melanoma. The radiation dose to the ciliary body and lens is lower with the CyberKnife method compared to Gamma Knife. However, the doses to the optic disc and macula are higher.^17^

In our study group, the globe salvage rate was 83.3%. In his pioneering paper, Muacevic et al.^18^ performed 18-22 Gy SRS on 20 patients with medium and large uveal melanoma and reported that none of the 7 patients they were able to follow up for more than 6 months required enucleation due to adverse effects or tumor growth. However, their case series was small, and the follow-up period was short. In a later paper from the same group, Eibl-Lindner et al.^19^ reported the results of 18-22 Gy SRS on 217 patients with medium or large uveal melanoma and reported a globe preservation rate of 86.7% at 3 years and 73% at 5 years.

In our series, the most common complications seen after SRS were cataract (38.9%) and radiation retinopathy (27.7%). In another publication from Turkey, Yazıcı et al.^20^ reported a 42.0% prevalence rate of radiation retinopathy in their 181-case series. The radiation dose to the critical intraocular structures including lens, optic disc, and macula depend on tumor location as well was the radiation method used. In our series, the rate of cataract formation was 38.9% and the tumor was in a peripapillary location in 60.0% of those patients. Radiation retinopathy was observed in 27.7% of the patients. There was a significant association between radiation retinopathy and distance of the tumor from the optic disc but not between radiation retinopathy and MRD to the tumor, tumor thickness, or distance from the fovea. Previous studies reported the distance of the tumor to the fovea as a risk factor for the development of radiation retinopathy.^21,22^ In our series, the mean distance of the tumor from the fovea was similar between patients who developed radiation retinopathy and those who did not. The similar mean values and small number of cases may explain why a statistically significant relationship was not detected.

Local recurrence is known to be associated with metastasis-related mortality.^23^ Recurrence was observed in 5 of the patients in our case series. Metastasis was observed in 2 of the patients who had recurrence and 1 of these patients died. Recurrence may occur due to problems with eye immobilization during SRS. Our globe salvage rate was 83.3% at a mean follow-up of 17.2 months.

As noninvasive methods, OCT and OCTA have made a significant contribution to the diagnosis and treatment of radiation maculopathy. OCT reveals macular thickening in the early stages of radiation maculopathy, followed by the development of cystoid macular edema. In advanced cases, subretinal fluid and opacities with increased reflectivity consistent with subretinal exudation and hemorrhage are observed on OCT.^24^

OCTA is a non-invasive angiography method that provides cross-sectional and volumetric information about the retina. While fluorescein angiography only allows evaluation of the superficial capillary plexus, OCTA enables separate imaging of the superficial and deep capillary plexuses, outer retina, and choriocapillaris layer. In radiation maculopathy, changes are observed in all four layers.^25^

According to our early results, SRS is an effective method for local control of uveal melanoma that provides patient comfort, saves time, and has a favorable adverse-effect profile. After SRS, patients should be followed closely for the development of radiation maculopathy with frequent OCT and OCTA imaging, and anti-VEGF therapy should be initiated at the onset of radiation maculopathy to improve visual prognosis. Laser photocoagulation can also be performed for retinal nonperfusion after wide-angle fluorescein angiography when necessary.

The main limitations of our study are that it is a retrospective study, it included a small number of cases, and the follow-up period was short. The safety of this treatment should be supported through studies with larger case series and longer follow-up periods.

## Figures and Tables

**Table 1:**
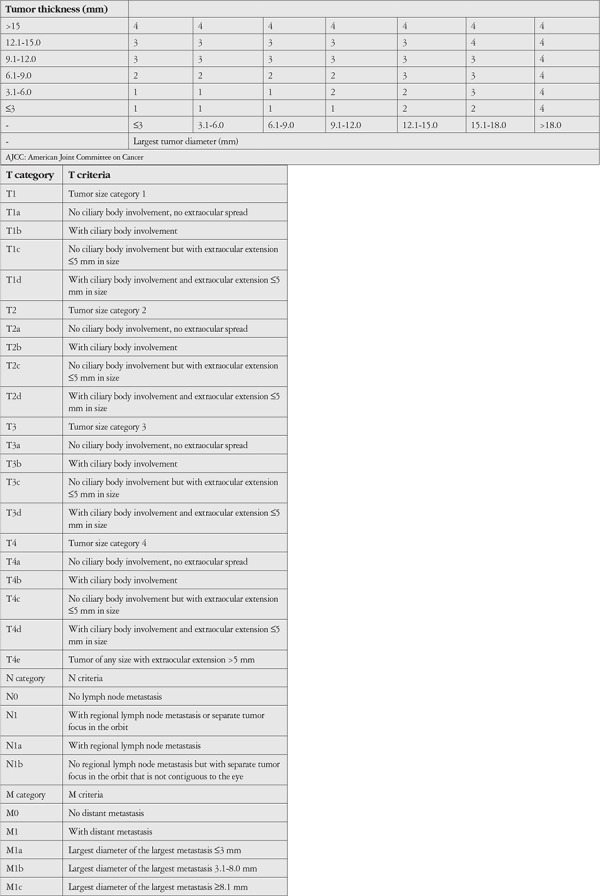
8^th^ Edition AJCC classification of posterior uvea melanoma

**Table 2:**
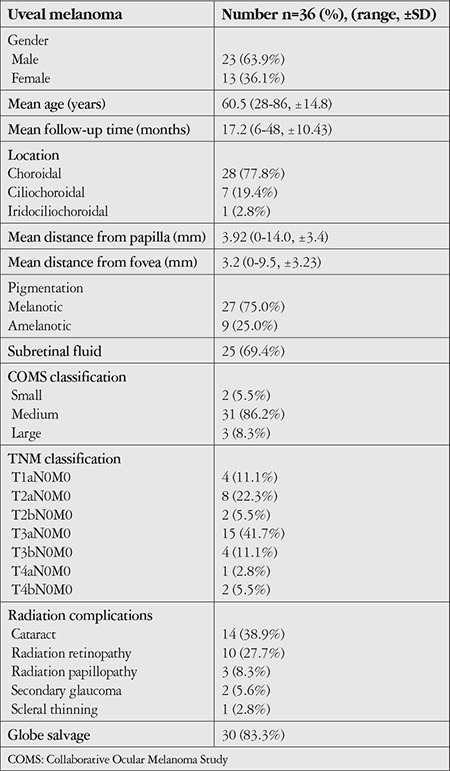
Characteristics of patients with uveal melanoma
